# Improved Salinity Tolerance in Carrizo Citrange Rootstock through Overexpression of Glyoxalase System Genes

**DOI:** 10.1155/2015/827951

**Published:** 2015-07-08

**Authors:** Ximena Alvarez-Gerding, Rowena Cortés-Bullemore, Consuelo Medina, Jesús L. Romero-Romero, Claudio Inostroza-Blancheteau, Felipe Aquea, Patricio Arce-Johnson

**Affiliations:** ^1^Departamento de Genética Molecular y Microbiología, Facultad de Ciencias Biológicas, Pontificia Universidad Católica de Chile, Avenida Alameda 340, P.O. Box 114-D, 8331150 Santiago, Chile; ^2^Facultad de Agronomía e Ingeniería Forestal, Pontificia Universidad Católica de Chile, Avenida Vicuña Mackenna 4560, 7820436 Santiago, Chile; ^3^Instituto Politécnico Nacional, CIIDIR, Unidad Sinaloa, Departamento de Biotecnología Agrícola, Boulevard Juan de Dios Bátiz Paredes No. 250, San Joachín, CP 81101, Guasave, SIN, Mexico; ^4^Núcleo de Investigación en Producción Alimentaria, Facultad de Recursos Naturales, Escuela de Agronomía, Universidad Católica de Temuco, P.O. Box 56-D, Temuco, Chile; ^5^Laboratorio de Bioingeniería, Facultad de Ingeniería y Ciencias, Universidad Adolfo Ibañez, Diagonal Las Torres 2640, Peñalolen, 7941169 Santiago, Chile; ^6^Center for Applied Ecology and Sustainability (CAPES), Santiago, Chile

## Abstract

Citrus plants are widely cultivated around the world and, however, are one of the most salt stress sensitive crops. To improve salinity tolerance, transgenic Carrizo citrange rootstocks that overexpress glyoxalase I and glyoxalase II genes were obtained and their salt stress tolerance was evaluated. Molecular analysis showed high expression for both glyoxalase genes (*BjGlyI* and *PgGlyII*) in 5H03 and 5H04 lines. Under control conditions, transgenic and wild type plants presented normal morphology. In salinity treatments, the transgenic plants showed less yellowing, marginal burn in lower leaves and showed less than 40% of leaf damage compared with wild type plants. The transgenic plants showed a significant increase in the dry weight of shoot but there are no differences in the root and complete plant dry weight. In addition, a higher accumulation of chlorine is observed in the roots in transgenic line 5H03 but in shoot it was lower. Also, the wild type plant accumulated around 20% more chlorine in the shoot compared to roots. These results suggest that heterologous expression of glyoxalase system genes could enhance salt stress tolerance in Carrizo citrange rootstock and could be a good biotechnological approach to improve the abiotic stress tolerance in woody plant species.

## 1. Introduction

Citrus are perennial plants that produce fruit with a high-value worldwide and are cultivated in tropical, subtropical, and Mediterranean climates. Drought and salinity are becoming particularly widespread in many regions and may cause serious salinization of more than 50% of all arable lands by the year 2050 [1]. High salinity can cause severe damage to plants leading to considerable reduction in their productivity. Lemons and oranges are the most salinity sensitive of all agricultural crops [2].

Salinity is mainly constituted by sodium chloride (NaCl) and high levels of salt exert its negative impact, mainly by disrupting the ionic and osmotic equilibrium of the cell, leading the plant growth inhibition and even death [3].

The ability of citrus trees to tolerate salinity varies among different species and depends on the rootstock. Carrizo citrange (*Citrus sinensis* L. Osb. x* Poncirus trifoliata* L. Raf.) is the most popular rootstock worldwide, because of its tolerance to diseases and flooding, moderate cold tolerance, good fruit quality, high yield, and good compatibility with varieties [4]. However, a major drawback is its sensitivity to salinity, which restricts its productive use [5]. A few citrus rootstock-breeding programs have succeeded in increasing salinity tolerance. In this context, genetic modification arises as an alternative to conventional breeding. Successful protocols for genetic transformation of Carrizo citrange have been developed over the past years [6]. To improve salinity tolerance, many alternatives have been described in annual species through gene transfer [7]. Among them, the overexpression of the glyoxalase system (glyoxalases I and II) has been demonstrated to be a good approach to enhance salinity tolerance in different plants like tobacco [8] and tomato [9]. This defense mechanism has been described which naturally exists in different plants. For example, the activity of glyoxalase I (Gly I) was increased in salt stress conditions in tolerant rice variety [10]. In addition, plants under high temperature induced an oxidative stress by simultaneously increasing nonenzymatic and enzymatic antioxidant responses, as well as detoxification systems for methylglyoxal (MG) [11].

In this work, we evaluate the response to salinity conditions of transgenic Carrizo citrange that overexpress two genes coding for the glyoxalase system (*BjGlyI* and* PgGlyII*) from* Brassica juncea *and* Pennisetum glaucum, *respectively.

## 2. Materials and Methods

### 2.1. Plant Material

Carrizo citrange seeds were soaked in a fungicide solution of Captan 1% (1 g/L) plus Benlate (0.8 g/L) in a shaker at 30°C for 48 h. Seeds were germinated in medium Murashige and Tucker solidified with 0.7% agar (w/v). To prevent contamination, 3 mL/L Plant Preservative Mixture (PPM, Plant Cell Technology) was added without sucrose. Seeds were then sown and placed in a germination chamber in darkness at 28 ± 2°C.

### 2.2. Genetic Constructions

**Figure 1:**
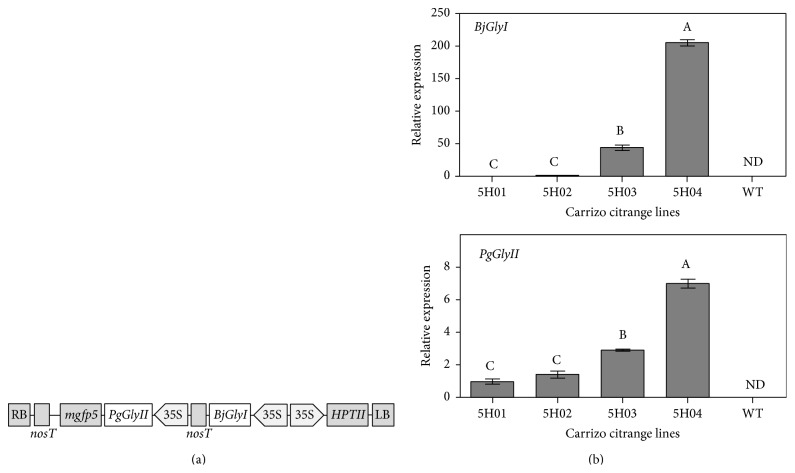
(a) Schematic representation of the T-DNA regions of the transformation vectors.* BjGlyI + PgGlyII* construction containing* glyoxalase I* from* Brassica juncea* (*GlyI*) and* glyoxalase II* from* Pennisetum glaucum* (*GlyII*) driven by independent CaMV35S promoters and containing the HPTII selectable marker gene. (b)* BjGlyI + PgGlyII *expression in Carrizo citrange transgenic lines. The transcript abundance was normalized to the lowest expression for each gene. Bars represent means of three plants ± standard error. Different uppercase letters within each figure and gene indicate significant differences at *P* ≤ 0.05. ND: not detected.

The double construct containing* BjGlyI* from* Brassica juncea* (GenBank accession number Y13239) and* PgGlyII* from* Pennisetum glaucum* (GenBank accession number AF508863.1) was driven by independent CaMV 35S promoters in the vector pCAMBIA1304 (Figure 1(a)).

### 2.3. Carrizo Citrange Transformation and Plant Regeneration

Epicotyl segments were transformed as described by Cervera et al. [6] using the* Agrobacterium tumefaciens* strain EHA105. Epicotyls were subsequently transferred to Petri plates containing hygromycin (3.5 mg/L) for selection. After three weeks in darkness, plates were transferred to a photoperiod of 16 h light and subcultured every two weeks in the same medium. Rooted explants were acclimated in hermetic transparent pots containing 50% perlite and 50% peat moss in a greenhouse and vegetatively propagated. Subsequently, after 12 weeks, plants were transferred to individual pots filled with sterile sand for salinity tolerance experiments.

### 2.4. Transgene Evaluation

Genomic DNA was isolated from leaf tissue using the Plant Genomic DNA extraction minikit (Favorgen Biotech Corp.). PCR amplifications of integrated transgenes in* BjGlyI* +* PgGlyII* lines were performed using primers* BjGlyI*-Fwd 5′-ATGGCGTCGGAAGCGAA-3′ and* BjGlyI*-Rev 5′-CGATCCAGTAGCCATCAG-3′ for glyoxalase I and* PgGlyII*-Fwd 5′-TTAAAAGTTATCCTTCGCTCG-3′ and* PgGlyII*-Rev 5′-ATGCGGATGCTGTCCAAGGCG-3′ for glyoxalase II, respectively. In addition, PCR genotyping was made using the primers 5′-TCGTCCATCACAGTTTGCC-3′ and 5′-AAAAGCCTGAACTCACCGC-3′ for hygromycin phosphotransferase protein (HPTII). The genotyping was performed initially in plants grown in vitro and then was repeated in the same plants grown in greenhouse conditions (2-month-old).

### 2.5. Quantitative Gene Expression

The RNA extraction, cDNA synthesis, and quantification of gene expression were performed as described previously [12]. Primers q*BjGlyI*-Fwd 5′-TGGGCATATTGGGGTTACAG-3′ and q*BjGlyI*-Rev 5′-CCATCGTGTGGTTTCTTGAC-3′; and q*PgGlyII*-Fwd 5′-ACGCTGCTCGTCATGCCTCT-3′ and q*PgGlyII* -Rev 5′-ATTTGCAGCGACGACGAGAC-3′ were used to analyze the expression of* BjGlyI *and* GlyII*, respectively. Primers were evaluated with conventional PCR to discard any endogenous amplification glyoxalase genes in wild type (WT) Carrizo citrange plants. Stratagene model Mx3000P and the SensimixTM SYBR-Green kit were used for qRT-PCR analysis and data was normalized using* Elongation Factor 1* [13].

### 2.6. Evaluation of Salinity Tolerance in* Carrizo Citrange* Plants

To evaluate salinity tolerance, Carrizo citrange transgenic and wild type plants were irrigated twice a week with Hoagland solution diluted to 25% v/v (control treatment) or Hoagland solution diluted to 25% v/v supplemented with 75 mM NaCl (saline treatment). After 30 days, plants were divided into shoots and roots and dried at 80°C for 72 h to measure dry weight (DW) and the Na^+^ and Cl^−^ concentration, which was conducted by the laboratories AgroLab Ltda., Santiago, Chile.

### 2.7. Statistical Analyses

Statistical analyses were performed with InfoStat software (http://www.infostat.com.ar). Experiments were carried out using a completely randomized design. Analysis of variance and covariance was applied depending on the trait. Tukey's (*P* ≤ 0.05) multiple comparison procedure was used when significant differences were found.

## 3. Results

### 3.1. Integration and Expression of Glyoxalase System Genes in Carrizo Citrange Transgenic Lines

Several transformation experiments to overexpress the glyoxalase system were carried out using epicotyls as explants. After a twelve-week period, hygromycin-resistant shoots were tested for transgene integration and expression by PCR and qRT-PCR analysis, respectively. The PCR genotyping was performed as described before in Materials and Methods (data not shown). Four transgenic lines that show transgene amplification and expressions of* BjGlyI* +* PgGlyII* (named 5H01, 5H02, 5H03, and 5H04) were chosen. The expression of transgenes was not detected in Carrizo citrange wild type plants (Figure 1(b)). For glyoxalase system transgenic lines, the highest expression for both* BjGlyI* and* PgGlyII* genes was observed in 5H03 and 5H04 in comparison with the transgenic lines 5H01 and 5H02 (Figure 1(b)). These genes are under the transcriptional control of 35S promotor. However, the* BjGlyI* gene has another 35S promoter upstream that control the expression of HPTII. The genetic architecture of the transgene can explain the huge difference in the level of expression of both transgenes inside the same line.

### 3.2. Tolerance of Transgenic Carrizo Citrange Plants to Moderate Salinity Levels

**Figure 2:**
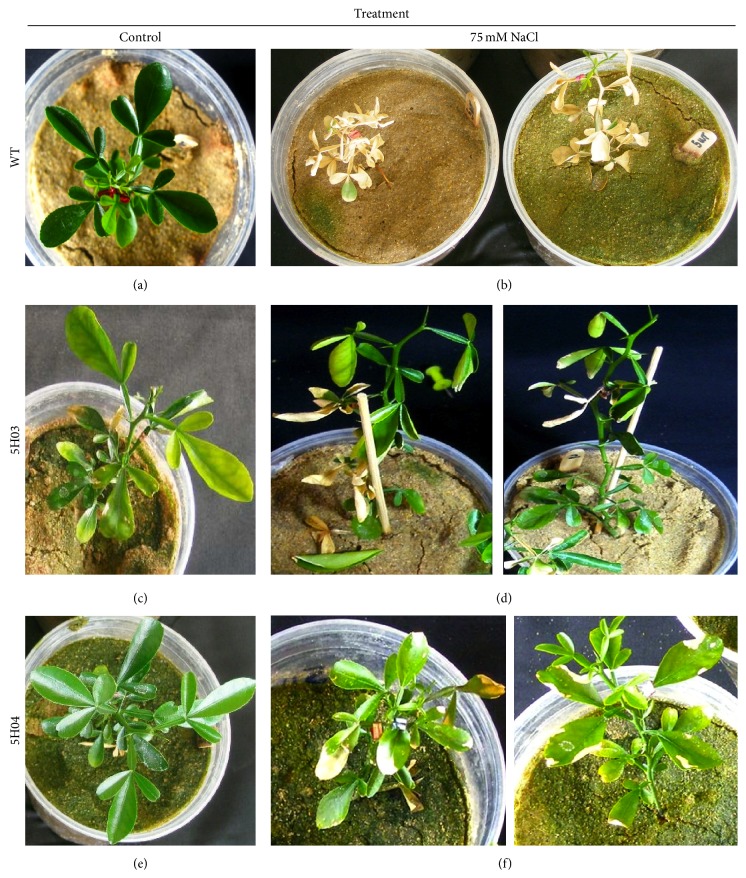
Citrus plant phenotypes after 30 d under 75 mM NaCl treatments. ((a), (c), and (e)) WT and transgenic plants under normal conditions. ((b), (d), and (f)) WT and transgenic plants under salinity stress treatment (75 mM NaCl).

To assess salinity tolerance of the transgenic Carrizo citrange rootstock, plants were irrigated with saline solutions to compare their response in qualitative and quantitative parameters. Under control conditions, transgenic and wild type plants presented normal plant and leaf morphology (Figures 2(a), 2(c), and 2(e)). When the lines were subjected to salinity treatments, more than 80% of wild type leaves became wilted (Figures 2(a) and 2(b)). In contrast, 5H03 and 5H04 transgenic lines showed less than 40% of leaf damage in the saline treatments (Figures 2(c)–2(f)).* BjGlyI* +* PgGlyII* transgenic lines 5H03 and 5H04 showed less yellowing and marginal burn in lower leaves under saline treatment. The other two transgenic lines 5H01 and 5H02 showed an intermediate phenotype (data not shown).

**Figure 3:**
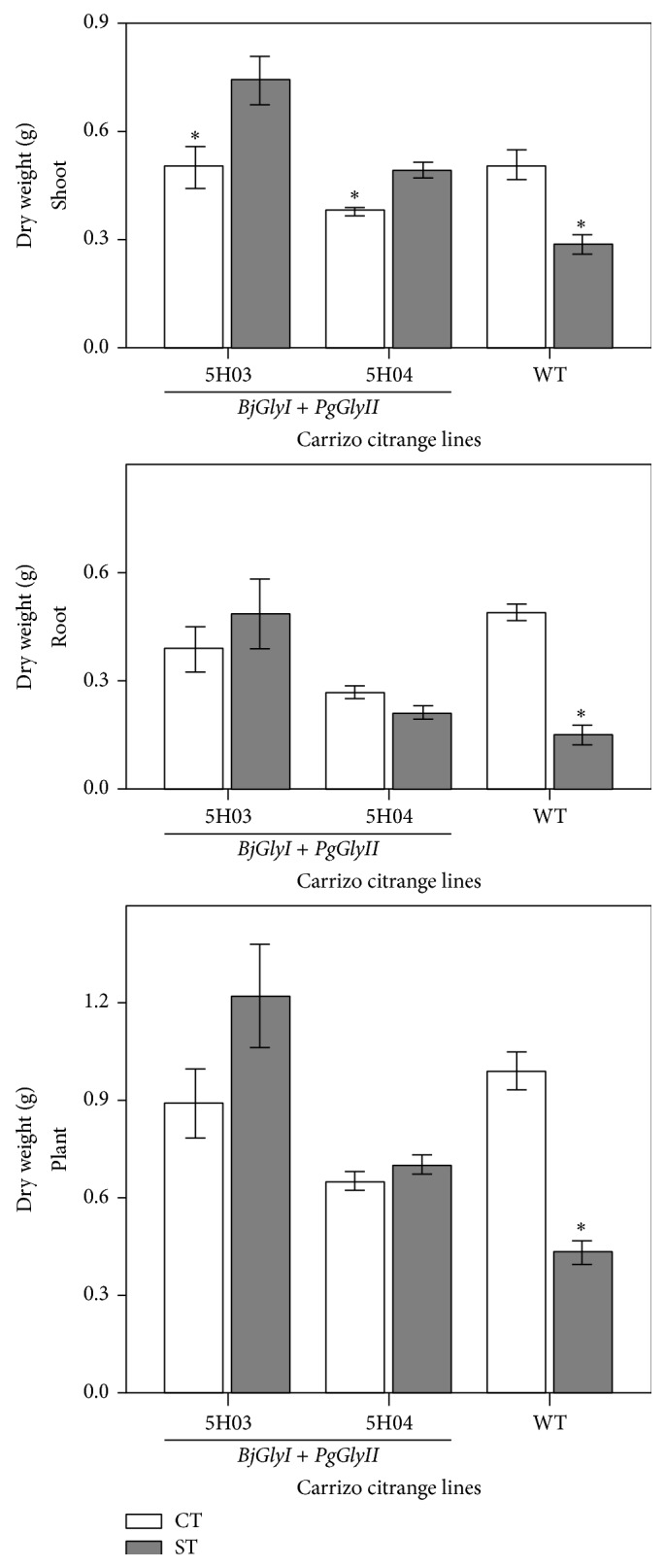
Dry weight of whole plants, shoots, and roots of WT and transgenic plants after a five-week salinity treatment. CT (control) and ST (salinity stress) treatments were compared by Student's* t*-test within each line.

With the purpose of quantifying how saline treatments affect growth, the dry weight (DW) of the shoot, root, and whole plant was measured. Significant differences in the DW were found in the tissues analyzed of the wild type plants (Figure 3). The DW of shoots, roots, and whole plants was decreased by 44%, 70%, and 57%, respectively, under saline treatment in wild type plants. The lines 5H03 and 5H04 showed no significant differences in the root and complete plant DW under saline treatment (Figure 3). However, there were significant differences in the shoot; lines 5H03 and 5H04 showed an increase in DW of 46% and 28%, respectively.

### 3.3. Chloride and Sodium Accumulation in Roots and Shoot

**Figure 4:**
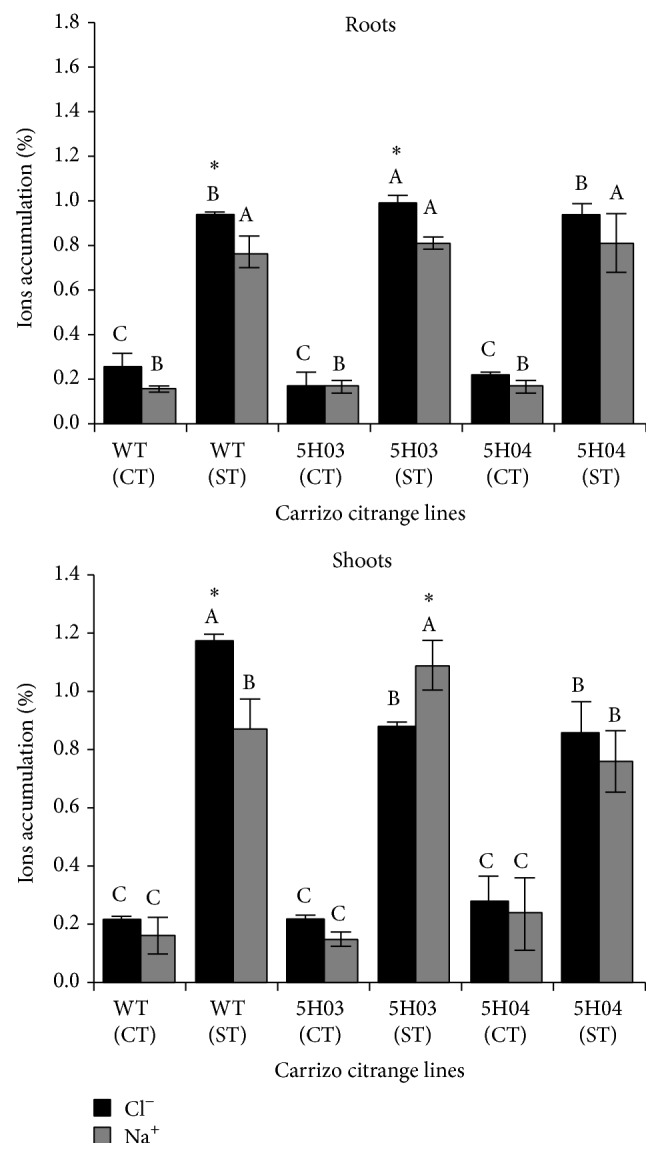
Effects of salt stress on the accumulation of chloride (Cl^−^) and sodium (Na^+^) in roots and shoots of Carrizo citrange rootstock under fresh water (control) and salinity (75 mM NaCl). The values represent averages of three replicates ± S.D. Different uppercase letters indicate significant differences (*P* ≤ 0.05) between lines in the same treatment. Asterisks indicate differences (*P* ≤ 0.05) between the treatments in the same line.

Salt treatment increased the accumulation of Cl^−^ and Na^+^ in roots and shoots in all lines (Figure 4). The accumulation of Cl^−^ was higher in roots of 5H03 transgenic lines but in shoots accumulation was lower. Also, a significant higher Na^+^ accumulation showed 5H03 transgenic line in this organ. No significant differences were observed between ions and organs in the 5H04 transgenic line. In addition, the WT line accumulated ~20% more in the shoots than roots (Figure 4).

## 4. Discussion 

Citrus plants are very sensitive to salinity, limiting their yield under this condition. Grafting onto salinity tolerance rootstocks is an alternative to overcome this problem. In this work, we report the transformation of Carrizo citrange rootstock with genes that codify for the glyoxalase system (*BjGlyI* and* PgGlyII*). For salinity tolerance assessment, plants were irrigated with NaCl under greenhouse conditions. The glyoxalase pathway might be operating through detoxification and cellular repair [8] leading to a better performance by decreasing oxidative stress, thus allowing higher dry mass accumulation as demonstrated by Singla-Pareek et al. [14] and Álvarez-Viveros et al. [9]. Specifically, the second enzyme of glyoxalase pathway (*GlyII*) that detoxifies cytotoxic metabolite methylglyoxal (MG) in salinity plant response contains a binuclear Zn/Fe centre in its active site and chelation that are essential for its activity [15].

Here, we evaluated the effect of overexpressing* BjGlyI* and* PgGlyII *in transgenic plants on biomass and phenotype under salinity stress. The transgenic 5H03 and 5H04 lines showed higher expression levels of both genes (Figure 1(b)). Shoot, root, and whole plant DW accumulation was reduced in Carrizo citrange WT by saline conditions, but not in 5H03 and 5H04 transgenic lines (Figure 3), which exhibited a healthy leaf phenotype (Figures 2(c)–2(f)). However, the shoot-to-root DW ratio under control conditions of* BjGlyI + PgGlyII* 5H03 line was similar to WT plants, indicating that both root and shoot kept growing (Figure 3). In* BjGlyI* +* PgGlyII *5H03 and 5H04 lines, root DWs were most affected by saline treatment. This may be associated with the vulnerability of the root to salinity as an indicator of salt accumulations in this zone [16]. In general, this is associated with the greater accumulation of Cl^−^ in roots (Figure 4), in line 5H03, and it is correlated with lower Cl^−^ accumulation and increased Na^+^ is observed in leaves of the 5H03 lines. Nonetheless, no significant differences were found between the ions in roots and leaves of the 5H04 line showing similar accumulation in both organs (Figure 4). However, the responses of 5H03 have been observed in other rootstocks, where plant growth is related not only to the osmotic effect, but also to a gradual accumulation of toxic levels of Cl^−^ and Na^+^ in the leaves [17]. In this case, the overexpression of* BjGlyI + PgGlyII* correlated positively with biomass and phenotype, suggesting that the double transgene glyoxalase system is more effective when coping with salinity stress in Carrizo citrange plants. In other plant systems, like* Pennisetum glaucum*, it has been showed that* GlyII* transcript levels are elevated during several abiotic stresses including anoxia, hypoxia, drought, light, and salt. In earlier studies, exposure of the plants to salt stress had led to increase in* GlyII* transcript levels of 15-fold increases at 24 h after treatment [18], which confirms that* GlyII* is induced during abiotic stress and suggests that it may have a biological role in stress tolerance [19]. The results of this work are according to these papers.

## 5. Conclusion

In this work, we report the overexpression of the glyoxalase system in citrus plants. This study provides direct evidence that constitutive overexpression of the* BjGlyI* +* PgGlyII* genes improve salinity tolerance of Carrizo citrus rootstock under greenhouse conditions. This work could provide insights into the benefits of transgenesis in woody plant species increasing salinity tolerance. However, further experiments are necessary under both greenhouse and field conditions that complement and lead to a better analysis of how the overexpression of these genes is acting to change rootstock and/or variety performance.
